# Supplementary material for: Analysis of Anti-Cancer and Anti-Inflammatory Properties of 25 High-THC Cannabis Extracts
Source: Molecules. 2022 Sep 16;27(18):6057. doi: 10.3390/molecules27186057 (PMC9506243; doi:10.3390/molecules27186057)
Supplement: Supplementary file 1 [file molecules-27-06057-s001.zip › molecules-1894412-supplementary.pdf]

Supplementary Figures

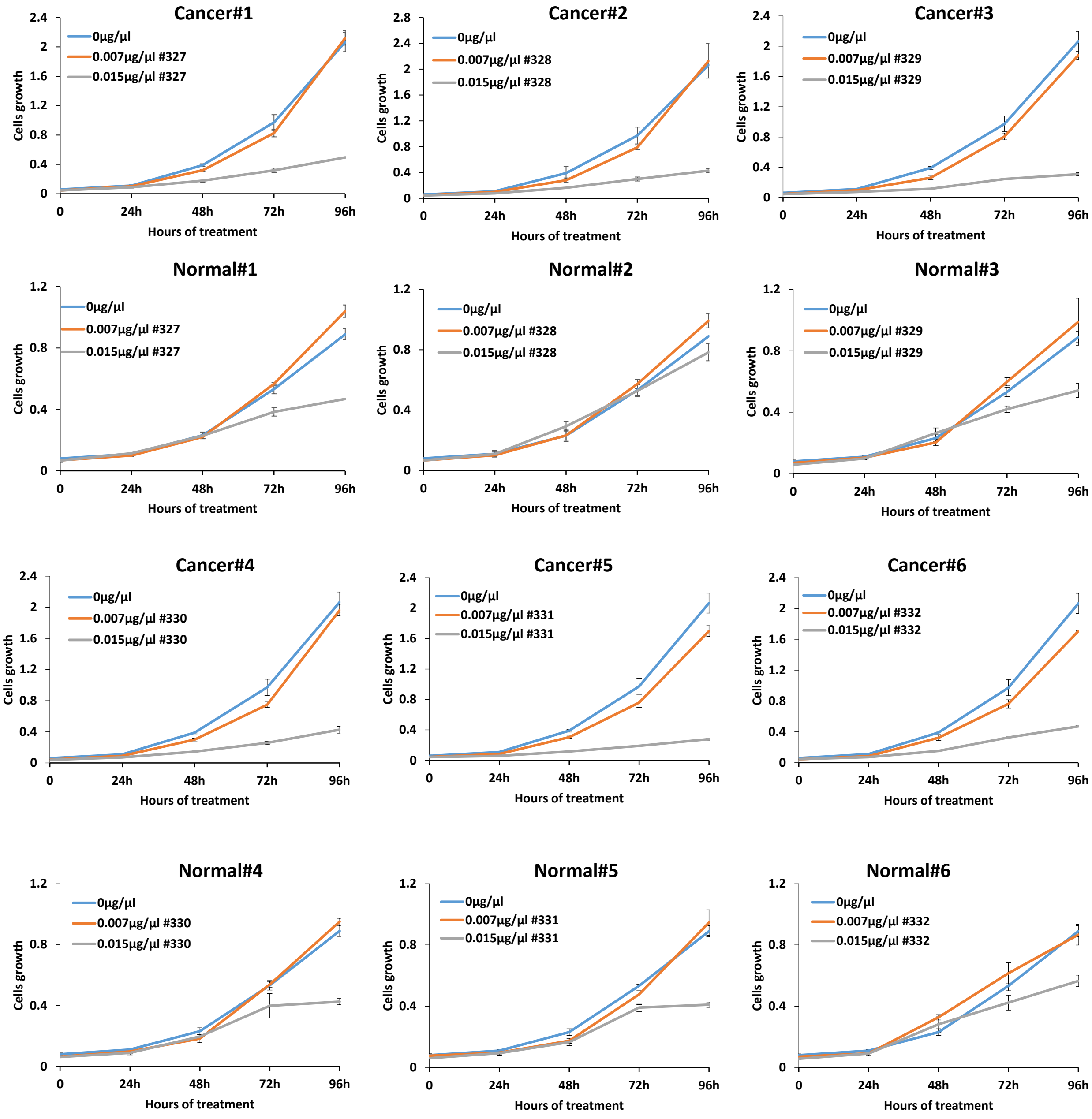

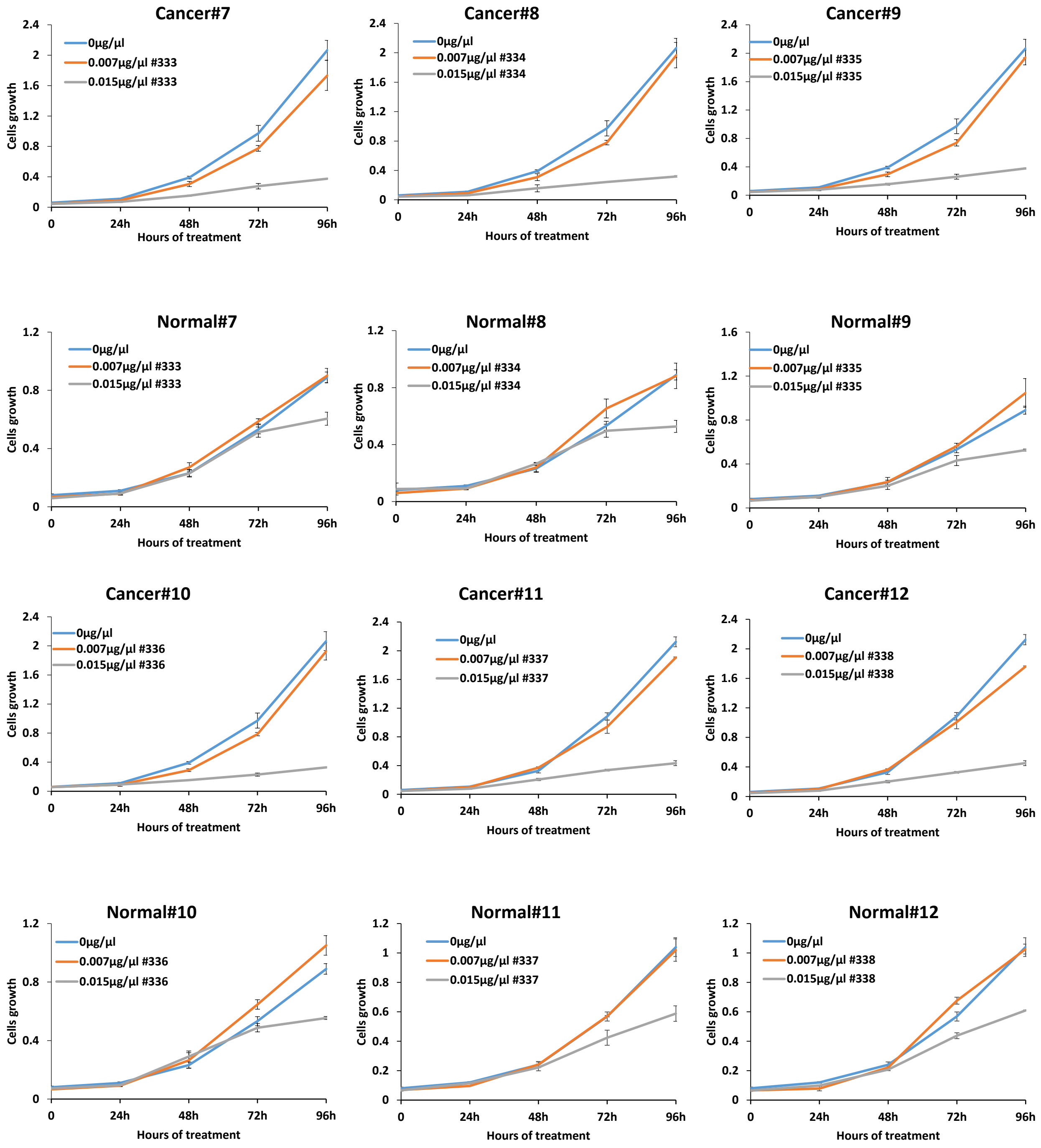
Cancer#7
0µg/µl
0.007µg/µl #333
0.015µg/µl #333
Cells growth
Hours of treatment
0 24h 48h 72h 96h
Cancer#8
0µg/µl
0.007µg/µl #334
0.015µg/µl #334
Cells growth
Hours of treatment
0 24h 48h 72h 96h
Cancer#9
0µg/µl
0.007µg/µl #335
0.015µg/µl #335
Cells growth
Hours of treatment
0 24h 48h 72h 96h
Normal#7
0µg/µl
0.007µg/µl #333
0.015µg/µl #333
Cells growth
Hours of treatment
0 24h 48h 72h 96h
Normal#8
0µg/µl
0.007µg/µl #334
0.015µg/µl #334
Cells growth
Hours of treatment
0 24h 48h 72h 96h
Normal#9
0µg/µl
0.007µg/µl #335
0.015µg/µl #335
Cells growth
Hours of treatment
0 24h 48h 72h 96h
Cancer#10
0µg/µl
0.007µg/µl #336
0.015µg/µl #336
Cells growth
Hours of treatment
0 24h 48h 72h 96h
Cancer#11
0µg/µl
0.007µg/µl #337
0.015µg/µl #337
Cells growth
Hours of treatment
0 24h 48h 72h 96h
Cancer#12
0µg/µl
0.007µg/µl #338
0.015µg/µl #338
Cells growth
Hours of treatment
0 24h 48h 72h 96h
Normal#10
0µg/µl
0.007µg/µl #336
0.015µg/µl #336
Cells growth
Hours of treatment
0 24h 48h 72h 96h
Normal#11
0µg/µl
0.007µg/µl #337
0.015µg/µl #337
Cells growth
Hours of treatment
0 24h 48h 72h 96h
Normal#12
0µg/µl
0.007µg/µl #338
0.015µg/µl #338
Cells growth
Hours of treatment
0 24h 48h 72h 96h

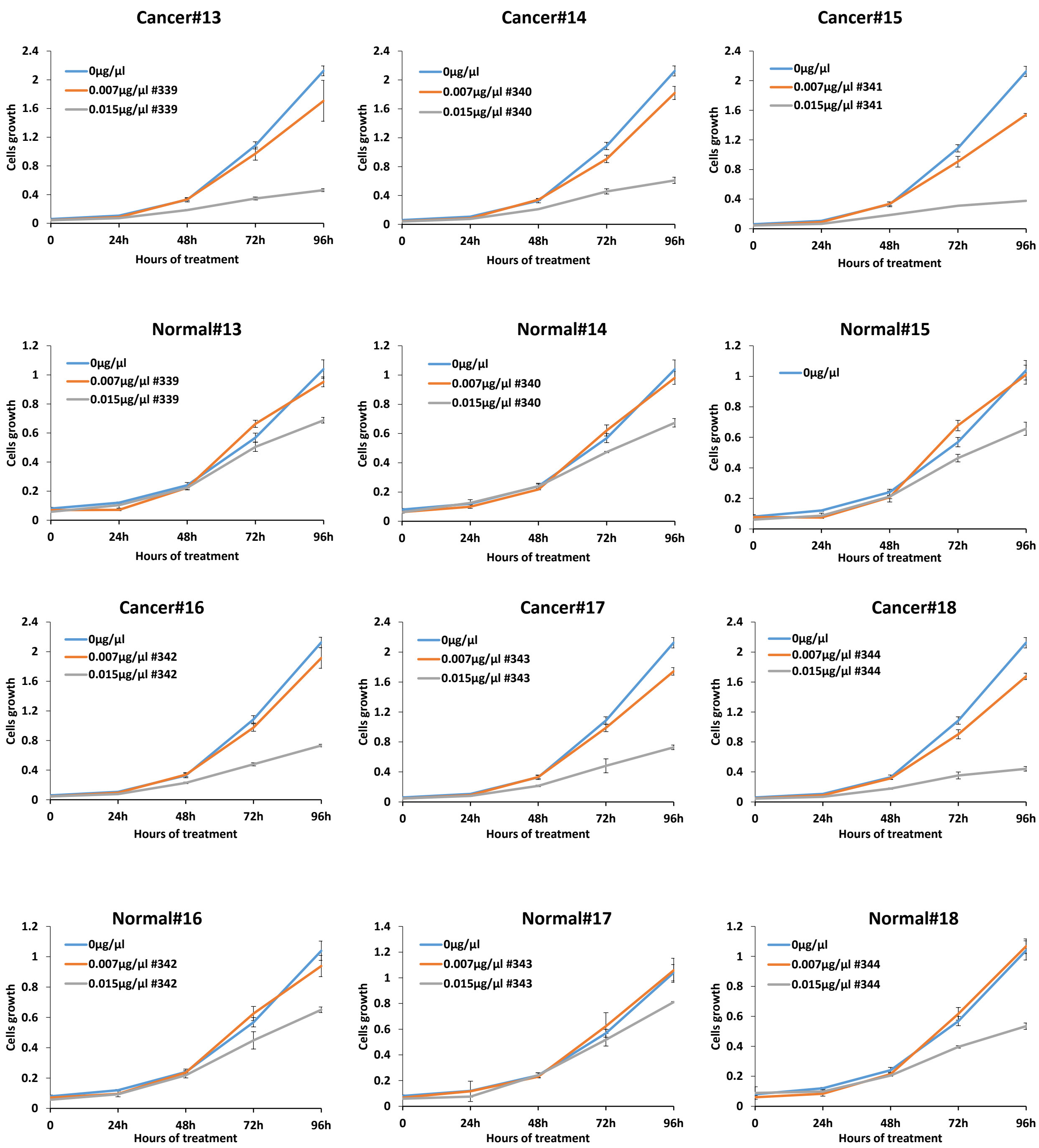

Cancer#13
0μg/μl
0.007μg/μl #339
0.015μg/μl #339
Cancer#14
0μg/μl
0.007μg/μl #340
0.015μg/μl #340
Cancer#15
0μg/μl
0.007μg/μl #341
0.015μg/μl #341
Normal#13
0μg/μl
0.007μg/μl #339
0.015μg/μl #339
Normal#14
0μg/μl
0.007μg/μl #340
0.015μg/μl #340
Normal#15
0μg/μl
Cancer#16
0μg/μl
0.007μg/μl #342
0.015μg/μl #342
Cancer#17
0μg/μl
0.007μg/μl #343
0.015μg/μl #343
Cancer#18
0μg/μl
0.007μg/μl #344
0.015μg/μl #344
Normal#16
0μg/μl
0.007μg/μl #342
0.015μg/μl #342
Normal#17
0μg/μl
0.007μg/μl #343
0.015μg/μl #343
Normal#18
0μg/μl
0.007μg/μl #344
0.015μg/μl #344
Cells growth
Hours of treatment
0
24h
48h
72h
96h

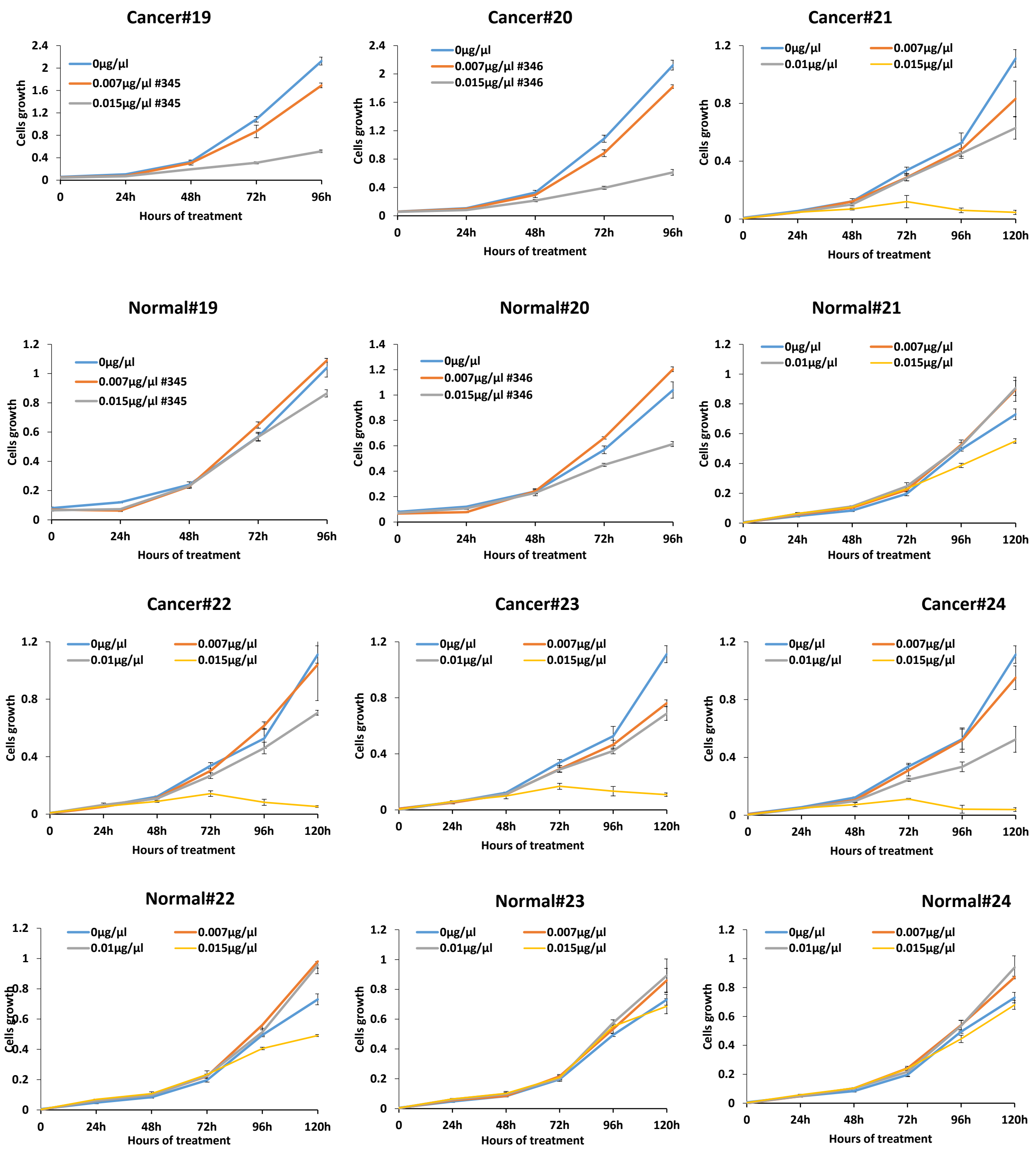
Cancer#19
0μg/μl
0.007μg/μl #345
0.015μg/μl #345
Cells growth
Hours of treatment
Cancer#20
0μg/μl
0.007μg/μl #346
0.015μg/μl #346
Cells growth
Hours of treatment
Cancer#21
0μg/μl
0.007μg/μl
0.01μg/μl
0.015μg/μl
Cells growth
Hours of treatment
Normal#19
0μg/μl
0.007μg/μl #345
0.015μg/μl #345
Cells growth
Hours of treatment
Normal#20
0μg/μl
0.007μg/μl #346
0.015μg/μl #346
Cells growth
Hours of treatment
Normal#21
0μg/μl
0.007μg/μl
0.01μg/μl
0.015μg/μl
Cells growth
Hours of treatment
Cancer#22
0μg/μl
0.007μg/μl
0.01μg/μl
0.015μg/μl
Cells growth
Hours of treatment
Cancer#23
0μg/μl
0.007μg/μl
0.01μg/μl
0.015μg/μl
Cells growth
Hours of treatment
Cancer#24
0μg/μl
0.007μg/μl
0.01μg/μl
0.015μg/μl
Cells growth
Hours of treatment
Normal#22
0μg/μl
0.007μg/μl
0.01μg/μl
0.015μg/μl
Cells growth
Hours of treatment
Normal#23
0μg/μl
0.007μg/μl
0.01μg/μl
0.015μg/μl
Cells growth
Hours of treatment
Normal#24
0μg/μl
0.007μg/μl
0.01μg/μl
0.015μg/μl
Cells growth
Hours of treatment

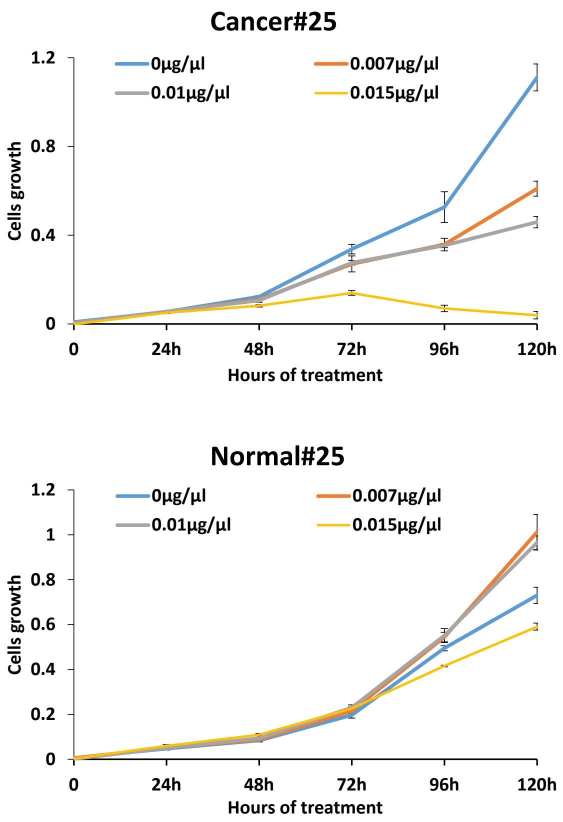

Supplementary Figure S1. Inhibition of growth of breast cancer cell line HCC1806 and normal cell line BJ-5ta. Y axis indicates arbitrary units of cell growth as measured by MTT assay. X axis indicates time of the cell growth/extraction application. Data (average of three replicates with SD) are shown as a cell growth curve measured for 96-120 h after application of three different concentrations of extracts #1-#25.

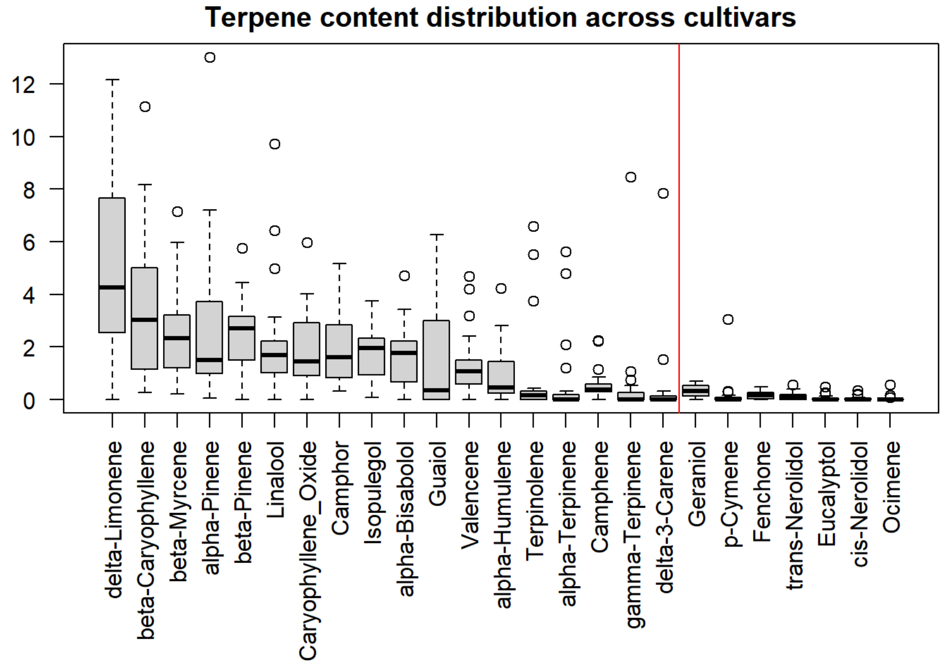

Supplementary Figure S2. Terpene content distribution across cultivars. Y axis shows the concentrations of terpenes in ug/ul. Data are shown as an average from 25 lines, with SD. Data outliers are shown as open circles. Terpenes to the right of the red line were removed from further analysis that included clustering and PCA.

Supplementary Tables

Supplementary Table S1. Enrichment of cannabinoids upon

**extraction**	**THC**	**CBD**	**CBGA**	**TOTAL**
**#1**	3.19	9.38	5.16	3.40
**#2**	4.13	10.33	5.86	4.29
**#3**	3.30	4.20	3.64	3.37
**#4**	3.17	3.84	3.45	3.23
**#5**	3.01	4.62	3.19	3.09
**#6**	2.89	2.83	2.35	2.86
**#7**	2.85	6.92	3.27	2.94
**#8**	3.12	3.44	2.73	3.14
**#9**	2.33	1.77	1.36	2.25
**#10**	2.33	5.09	2.31	2.40
**#11**	4.39	3.57	4.06	4.32
**#12**	3.04	3.55	3.09	3.05
**#13**	3.33	4.75	2.40	3.31
**#14**	2.76	7.55	2.77	2.84
**#15**	2.45	3.39	1.94	2.46
**#16**	2.76	4.24	4.96	2.87
**#17**	1.95	3.18	1.62	1.98
**#18**	2.85	4.31	3.03	2.91
**#19**	3.31	1.14	1.91	3.14
**#20**	2.91	8.98	4.19	3.06
**#21**	2.54	2.17	1.59	2.45
**#22**	2.83	3.22	2.10	2.80
**#23**	3.52	8.14	3.14	3.58
**#24**	2.31	1.57	1.41	2.20
**#25**	2.21	8.12	2.79	2.28
**Average**	**2.94**	**4.81**	**2.97**	**2.97**

Numbers show the enrichment of each tested cannabinoid, calculated by dividing the concentration in the extracts by the concentration in the flowers.

Supplementary Table S2. Concentration of terpenes in the flower extracts, ug/ul

	#1	#2	#3	#4	#5	#6	#7	#8	#9	#10	#11	#12	#13
δ-Limonene	8.06	3.76	4.12	6.36	9.68	4.25	4.33	0	12.15	7.65	0.88	5.45	8.36
β-Myrcene	5.98	0.82	0.79	0.52	2.79	2.65	0.85	3.78	1.21	7.16	5.67	2.1	2.07
α-Pinene	3.11	0.69	7.2	13.01	2.31	0.55	0.86	6.86	4.75	1.61	3.85	1.01	1.52
β-Caryophyllene	2.1	11.1	0.74	0.93	2.52	8.15	0.85	2.87	5.05	3.73	5.08	8.18	1.63
β-Pinene	1.87	0.92	3.44	5.75	2.7	0.92	4.33	0	3.15	1.84	3.99	2.99	1.5
Camphor	1.62	2.52	1.55	2.95	0.82	2.3	0.31	0.4	2.13	0.76	2.97	1.54	1.11
Isopulegol	0.81	3.43	1.45	1.75	2.13	3.46	0.45	2.54	3.75	2.24	1.96	2.54	1.26
Valencene	0.79	4.68	0.2	0.45	0.5	3.19	0	1.07	1.5	0.83	1.38	2.04	0.68
Geraniol	0.59	0.61	0	0.55	0	0.31	0	0.47	0.31	0	0.1	0.13	0.21
Camphene	0.52	0.32	0.33	0.31	0.86	0.25	0.34	2.26	1.16	0.62	0.07	0.37	0.59
Caryophyllene Oxide	0.49	5.98	1.24	0.97	1.45	2.91	0.7	0	2.47	3	2.59	3.22	0.24
Terpinolene	0.42	0	0.28	0	0.36	0.22	0	0	0.32	0.19	0.07	0.17	0.38
Eucalyptol	0.27	0	0.23	0	0	0	0	0	0.12	0	0	0	0
trans-Nerolidol	0.09	0	0.09	0	0	0.18	0	0	0.23	0	0.56	0.04	0.1
α-Bisabolol	0	4.71	1.38	1.18	0.68	2.73	0.31	1.78	2.63	2.22	1.44	1.1	0.68
α-Humulene	0	4.24	0.36	0	0	1.72	0	0.23	0.47	0.35	1.44	2.8	0.31
α-Terpinene	0	0.2	0	0.32	0	0	0	0	0	0	0	0.11	0
cis-Nerolidol	0	0	0	0	0	0	0	0	0	0	0.04	0.08	0
δ-3-Carene	0	0	0	0	0.3	0	0	7.84	0	0	0	0	0
Fenchone	0	0.41	0.48	0.41	0.34	0.13	0.18	0	0.21	0.36	0.08	0.06	0.26
γ-Terpinene	0	0	0.53	0	0	0	0	0	1.08	0	0	0	0
Guaiol	0	0.27	0.67	0	0	3.74	0	3.53	6.28	0	0.06	0.07	0.65
Linalool	0	2.22	1.09	0	3.14	1.53	1.26	4.98	6.43	1.8	1.2	1.7	2
Ocimene	0	0	0	0.56	0	0	0	0	0	0	0	0	0
p-Cymene	0	0	0	0.3	0	0	0	0	0	0	0.08	0.09	0
**Total**	**26.72**	**46.92**	**25.77**	**38.23**	**30.58**	**39.19**	**12.15**	**38.61**	**55.4**	**34.36**	**33.51**	**35.79**	**23.55**
	**#14**	**#15**	**#16**	**#17**	**#18**	**#19**	**#20**	**#21**	**#22**	**#23**	**#24**	**#25**	
δ-Limonene	3.6	8.68		8.44	4.97	4.27	4.99	1.01	0.58	2.55	2.78	0	
β-Myrcene	0.99	3.01	2.32	3.19	2.12	1.27	2.93	0.21	5.92	3.22	4.08	2.04	
α-Pinene	3.72	4.79	0.63	1.3	1.3	0.05	0.82	1.37	1.69	0.99	1.5	1.23	
β-Caryophyllene	0.83	3.87	4.55	7.62	3.53	3.02	4.08	0.28	1.14	1.01	1.25	5	
β-Pinene	0.92	1.81	2.25	4.36	2.58	3.03	1.19	0.15	3.14	2.93	2.8	4.44	
Camphor	1.24	3.07	0.75	1.66	3.88	5.16	1.93	2.85	0.6	3.25	0.78	1.25	
Isopulegol	0.8	2.52	1.95	0.99	1.15	1.99	2.34	2.32	0.08	0.33	0.54	0.93	
Valencene	0	4.21	2.41	1.21	1.34	0.72	1.99	0.68	0	1.13	0.59	1.36	
Geraniol	0	0.46	0.14	0.46	0.57	0.67	0.19	0.63	0.14	0.7	0.13	0.35	
Camphene	0.24	0.45	0.37	0.61	0.09	0.6	0.3	2.22	0.05	0	0.46	0.56	

Caryophyllene Oxide	0.81	2.78	1.31	3.53	0.39	1.74	4.01	1.28	1.16	0.92	1.67	3.37	
Terpinolene	0	0.09	0.07	0.2	0.01	0	0	3.76	6.59	5.53	0.28	0.12	
Eucalyptol	0	0		0.13	0	0	0	0	0.06	0.06	0.48	0.05	
trans-Nerolidol	0	0.05	0.41	0.01	0.36	0.18	0.35	0.56	0.07	0.14	0.02	0.12	
α-Bisabolol	0.42	2.15	2.25	1.01	3.44	2.2	1.96	2.11	0.56	0.36	2.28	1.89	
α-Humulene	0.24	1.1	1.93	0.51	1.41	1.5	2.42	1.12	0	0.51	0.06	0.27	
α-Terpinene	0	0	4.79	0	0	0.2	0.11	2.1	1.2	0	0	5.63	
cis-Nerolidol	0	0	0.06	0.08	0.06	0.18	0.22	0.34	0.02	0.02	0.03	0.02	
δ-3-Carene	0	0	0.03	0	0.24	0	0	1.54	0.32	0.13	0.2	0	
Fenchone	0	0.16		0.12	0.07	0.23	0	0.23	0.02	0.16	0.29	0.03	
γ-Terpinene	0	0.12		0	0.42	0.28	0	0.22	0.26	0.76	8.47	0	
Guaiol	0.39	0	2.72	0.03	4.04	5.07	0	3.07	1.17	0.09	2.99	0.35	
Linalool	1.01	9.73	2.48	2.78	0.65	1.73	0.51	2.12	0	0.84	1.31	2.17	
Ocimene	0	0		0	0	0	0	0	0	0.09	0.16	0	
p-Cymene	0.29	0	0.02	0.06	0	0	0	0.32	3.06	0	0	0.15	
**Total**	**15.5**	**49.05**	**31.44**	**38.3**	**32.62**	**34.09**	**30.34**	**30.49**	**28.17**	**25.72**	**33.15**	**31.33**	

Supplementary Table S3. CCE for various clustering methods

Clustering Method	Raw data	Scaled	Scaled and filtered
Ward.D2	0.46	0.65	0.66
Complete	0.74	0.71	0.72
McQuitty	0.77	0.85	0.86

Supplementary Table S4. Dunn index for various clustering methods

Clustering Method	Raw data	Scaled	Scaled and filtered
Ward.D2	0.33	0.37	0.36
Complete	0.29	0.45	0.44
McQuitty	0.38	0.60	0.64

Supplementary Table S5. Association between terpene content and anti-cancer activity

**Terpene**	**Estimate**	**Std. Error**	**t value**	**Pr(>|t|)**	**Pval_BH**

**γ-terpinene**	0.872	0.180	4.835	0.000*	0.002*
**eucalyptol**	9.558	3.176	3.010	0.006	0.094
**β-caryophyllene**	-0.240	0.145	-1.653	0.112	0.696
**camphene**	1.298	0.712	1.823	0.081	0.696
**α-humulene**	-0.627	0.384	-1.634	0.116	0.696
**CBGA**	1.512	1.171	1.291	0.210	0.699
**d-limonene**	-0.172	0.128	-1.345	0.192	0.699
**camphor**	-0.456	0.346	-1.318	0.201	0.699
**fenchone**	4.168	2.822	1.477	0.154	0.699
**valencene**	-0.413	0.344	-1.199	0.243	0.728

Asterisks show significant associations between the presence of terpenes and anti-cancer activity. The analysis was done using generalized linear models (GLM) with family "gaussian" specified. Resulting p-values were adjusted using Benjamini-Hochberg method.

Supplementary Table S6. Association between terpene or cannabinoids content and IL-6 expression

**Terpene**	**Estimate**	**Std. Error**	**t value**	**Pr(>|t|)**	**Pval_BH**
**p-cymene**	0.663	0.191	3.463	0.002*	0.063*
**camphor**	-0.297	0.102	-2.907	0.008*	0.119
**β-myrcene**	0.177	0.067	2.627	0.015*	0.151
**cis_nerolidol**	-3.143	1.594	-1.971	0.061	0.456
**CBD**	-0.817	0.566	-1.443	0.162	0.542
**geraniol**	-0.817	0.566	-1.443	0.162	0.542
**terpinolene**	0.119	0.078	1.530	0.140	0.542
**eucalyptol**	1.763	1.193	1.478	0.153	0.542
**trans-nerolidol**	-1.296	0.781	-1.659	0.111	0.542
**CBGA**	0.401	0.395	1.015	0.321	0.812

Asterisks show significant associations between the presence of terpenes or cannabinoids and the change in expression of IL-6. The analysis was done using generalized linear models (GLM) with family "gaussian" specified. Resulting p-values were adjusted using Benjamini-Hochberg method.

Supplementary Table S7. Association between cannabinoids content and COX2 expression

Terpene	Estimate	Std. Error	t value	Pr(>|t|)	Pval_BH
**trans-nerolidol**	-0.789	0.431	-1.830	0.080	0.948
**caryophyllene oxide**	0.084	0.054	1.560	0.132	0.948
**β-pinene**	0.061	0.055	1.118	0.275	0.948
**eucalyptol**	0.735	0.681	1.080	0.291	0.948
**fenchone**	0.575	0.545	1.056	0.303	0.948
**β-myrcene**	-0.044	0.042	-1.047	0.306	0.948
**α-bisabolol**	0.067	0.072	0.932	0.361	0.948
**cis_nerolidol**	-0.802	0.949	-0.845	0.407	0.948
**β-caryophyllene**	0.023	0.028	0.825	0.418	0.948
**geraniol**	0.257	0.326	0.788	0.439	0.948

Associations were done between the presence of terpenes or cannabinoids and the change in expression of COX2. The analysis was done using generalized linear models (GLM) with family "gaussian" specified. Resulting p-values were adjusted using Benjamini-Hochberg method.

Supplementary Table S8. Association between terpene and cannabinoids content

Terpene	Estimate	Std. Error	t value	Pr(>|t|)	Pval_BH
**α-pinene vs CBD**	0.158	0.04	4.005	0.001	0.014
**γ-terpinene vs CBGA**	0.133	0.032	4.216	0.000	0.008
**linalool vs CBGA**	0.358	0.096	3.723	0.001	0.014

Associations was done between the presence of terpenes and cannabinoids content and the change in expression of IL-6. The analysis was done using generalized linear models (GLM) with family "gaussian" specified. Resulting p-values were adjusted using Benjamini-Hochberg method.

Supplementary Table S9. List of cultivars used for this study

	Cultivar	Location	Source
1	CB14 (no commercial name)	Squamish	Cloudburst Cannabis

2	CB2 (no commercial name)	Squamish	Cloudburst Cannabis
3	CB24 (no commercial name)	Squamish	Cloudburst Cannabis
4	CB10 (no commercial name)	Squamish	Cloudburst Cannabis
5	CB19 (no commercial name)	Squamish	Cloudburst Cannabis
6	Emperior	Nelson	Cannabis West Development
7	Artic Kush	Nelson	Cannabis West Development
8	Elevator Kush	Nelson	Cannabis West Development
9	Sunshine Pie	Nelson	Cannabis West Development
10	Diamond Haze	Nelson	Cannabis West Development
11	Spaceman Plus	Nelson	Cannabis West Development
12	Florida Gold	Nelson	Cannabis West Development
13	Citrus Splash	Nelson	Cannabis West Development
14	Glacier Goo	Nelson	Cannabis West Development
15	Zombie	Nelson	Cannabis West Development
16	Berry Delight	Nelson	Cannabis West Development
17	Aviator	Nelson	Cannabis West Development
18	CB17 (Mom) (no commercial name)	Squamish	Cloudburst Cannabis
19	CB17 (regular)(no commercial name)	Squamish	Cloudburst Cannabis
20	CB3 (no commercial name)	Squamish	Cloudburst Cannabis
21	CB11 (no commercial name)	Squamish	Cloudburst Cannabis
22	CB13 (no commercial name)	Squamish	Cloudburst Cannabis
23	CB5 (no commercial name)	Squamish	Cloudburst Cannabis
24	CB9 (no commercial name)	Squamish	Cloudburst Cannabis
25	CB22 (no commercial name)	Squamish	Cloudburst Cannabis
